# Examining and Comparing the Energy Expenditure of Two Modes of a Virtual Reality Fitness Game (Supernatural): Indirect Calorimetry Study

**DOI:** 10.2196/53999

**Published:** 2024-06-04

**Authors:** Tabitha V Craig, Ryan E Rhodes, Wuyou Sui

**Affiliations:** 1 Department of Exercise Science, Physical & Health Education University of Victoria Victoria, BC Canada; 2 Behavioural Medicine Lab Department of Exercise Science, Physical & Health Education University of Victoria Victoria, BC Canada

**Keywords:** energy expenditure, exergaming, indirect calorimetry, virtual reality, VR, VR fitness, VR gaming

## Abstract

**Background:**

The effectiveness of virtual reality (VR) fitness games as a form of moderate to vigorous physical activity has yet to be thoroughly quantified through gold standard energy expenditure measures.

**Objective:**

The purpose of this study was to examine the energy expenditure of 2 medium-intensity modes (“Flow and “Boxing”) of a VR fitness game, Supernatural, using indirect calorimetry.

**Methods:**

Indirect calorimetry was used to examine relative and objective maximal oxygen consumption (VO_2_ max), metabolic equivalents of task (METs), and calories burned during medium-intensity bouts of both Flow and Boxing gameplay modes in young (mean age 25.42, SD 3.25 years), active individuals (n=12 female and n=11 male). METs and calories were also compared using a triaxial waist-worn accelerometer, an Apple smartwatch, and a VR headset. Mood states were assessed pre- and postbout using the shortened Profile of Mood States Questionnaire*.* Paired 2-tailed *t* tests were used to examine differences in game modes, between sexes, and pre-post exercise sessions.

**Results:**

Objective and relative VO_2_ max averaged 1.93 (SD 0.44) L/min and 27.61 (SD 5.60) mL/kg/min, respectively, between modes. Flow (mean 8.2, SD 1.54 METs) and Boxing (mean 7.6, SD 1.66 METs) are both classified as high energy expenditure, vigorous activities. Calorie expenditure data of the accelerometer and VR headset differed significantly from the metabolic cart. Mood changes pre- to post exercise were consistent with expected values for moderate- to vigorous-intensity physical activity, with participants reporting that they felt more “active,” “full of pep,” “vigorous,” and “lively” (*P*<.05) following bouts. Male individuals reported higher objective oxygen consumption (VO_2_) for both Flow and Boxing modes; no other sex-specific differences were observed.

**Conclusions:**

Both Flow and Boxing gameplay modes of Supernatural classify as vigorous physical activity and demonstrate the potential to promote mental and physical health benefits. Supernatural may be an effective exercise modality in a VO_2_ training program.

## Introduction

Regular physical activity (PA), described as a movement-driven increase in energy expenditure [1], is an important health behavior that has well-established mental and physical benefits [2]. Evidence suggests that individuals who partake in regular PA of 150 minutes of moderate-intensity PA or 75-150 minutes of moderate- to vigorous-intensity PA (MVPA) each week [3] have a lower risk of cancer, type 2 diabetes mellitus, ischemic heart disease, and ischemic stroke [4].

While there are diverse modes in which individuals can engage in regular PA, at-home exercise options have increased in popularity recently [5]. Before the COVID-19 pandemic, at-home PA may have been chosen due to the ease of access, high levels of autonomy in modifying both workouts and equipment, and the increased feeling of comfort provided by exercising in a familiar environment [6]. Due to COVID-19–related shutdowns of traditionally frequented centers for PA (eg, gyms, recreation centers, and organized sports leagues), individuals had to develop new routines to stay active. Many turned to home fitness workouts and exergames, including virtual reality (VR) fitness options, to maintain their PA levels [7,8]. Even beyond the pandemic, home workout options, such as VR gaming and internet-based fitness workouts, continue to appeal to individuals, in part due to their convenience and accessibility.

VR can be described as a head-mounted amalgamation of the human perception of sensation into an interactive virtual environment [9]. Contemporary iterations of VR, specifically immersive VR (such as the Oculus Quest 2 or HTC Vive), involve a head-mounted device inlaid with cameras that track the wearer’s environment, in order to project a virtual environment within the headset. VR gaming (also known as VR exercising, VR game exercising, or active VR games) refers to the use of a VR headset (and often handheld controllers) to engage in a single immersive, digital fitness experience consisting of both video game and exercise elements [10]. VR gaming differs from traditional exergaming (eg, Wii Fit and Just Dance) in that the gaming environment is visually immersive and interactive, rather than having visuals confined to a screen and movements or interactivity restricted by the fidelity of an external camera. These limitations of exergaming may contribute to their variability in energy expenditure [11]. Popular VR games include Beat Saber (Beat Games), Fruit Ninja VR (Halfbrick Studios), HOLOFIT (Holodia Holofit), Dance Central (Harmonix Music Systems), and BOXVR (FitXR). VR fitness demonstrated both mental and physical health during the COVID-19 lockdown [12]. Thus, VR fitness continues to remain popular as we emerge from the era of COVID-19 lockdowns, in part due to its enjoyable, flexible, and motivating nature [13].

While VR is gaining in popularity as a form of PA, there are, unfortunately, several factors that currently limit our understanding of whether it can serve as an effective means of achieving MVPA intensity. There is a tremendous amount of variance in the equipment involved within a VR gaming setup. Many studies use specialized equipment (eg, cycle ergometer [14], bespoke machinery [15], or sport-specific VR setups [16]), which would be unrealistic or inaccessible for most users to acquire or comfortably use. Among studies that examined a “regular” VR gaming setup, other confounding factors may also be at play. For example, some studies examine the use of additional accessories to existing VR setups, such as hand weights [17], which further obfuscate the energy expenditure of VR games.

Even in previous research that has used more consumer-friendly VR (eg, Oculus Quest and HTC Vive), the VR games themselves are quite variable in what they demand from the user. For example, Beat Saber, a popular VR rhythm game, requires users to hit targets in a virtual space with either one or both of their handheld controllers. However, the intensity with which these targets must be hit to register as a valid hit can be quite low. Additionally, the movements facilitated by VR games can vary, leading to a change in energy expenditure. Stewart [18] compared the energy expenditure from 3 different VR games, including Beat Saber, and found that each game demonstrated a significantly different energy expenditure for a similar playtime. Given the nature of VR setups and the range of movement they require [19], few studies have used the gold-standard measures of energy expenditure (eg, indirect calorimetry) within VR gaming studies. Furthermore, the presence of sex and gender differences within the field of VR gaming remains a controversial topic, with mixed evidence supporting a higher rate of physical discomfort and symptoms for female individuals [20,21]. Combined with the tendency for male individuals to demonstrate higher maximal oxygen consumption (VO_2_ max) values when controlling for age [22], investigating the presence of any sex-specific differences within VR gaming is of importance.

Supernatural is a VR fitness service that is available on the Oculus Quest (Meta) headsets, a popular consumer VR headset. Supernatural was chosen due to its specificity as a fitness service [23]. Other contemporary titles, like Beat Saber and Fruit Ninja VR, play closer to rhythm or arcade games in that the goal of the game is to achieve a high score, which can be done by hitting more targets. Supernatural is similar in that target accuracy is a metric; however, the key difference is that Supernatural records a metric of movement power (ie, power for the “Flow” mode and speed for the “Boxing” mode). In this way, Supernatural provides a means to compare effort, not just accuracy. Hence, limiting our study to just Supernatural was deemed appropriate. Thus, the primary purpose of this study was to examine and compare the energy expenditure of a bout of the VR fitness game Supernatural through indirect calorimetry; specifically, we examined the energy expenditure of both the Flow and Boxing modes, selecting workout durations and intensities (ie, medium intensity) based on the game’s average workout data. Comparisons between sexes (ie, male and female) were also examined as part of this primary objective.

The emerging popularity and novelty of VR gaming presents a further point of interest insofar as how accurate traditional PA monitoring devices are when applied to VR contexts. Traditional devices, such as waist-worn accelerometers and wrist-worn accelerometers (eg, smartwatches), demonstrate reasonable validity for capturing walking and running behaviors [24]; however, whether this extends to the more space-restricted, calisthenic-focused movements of VR gaming is unclear [25,26]. The inclusion of an onboard accelerometer in many consumer VR headsets is also a noteworthy activity monitor that is worth comparing to gold-standard energy expenditure measures [23]. Thus, the secondary purpose of this study was to examine the energy expenditure of the VR game as measured by accelerometry, consumer activity monitor (ie, Apple Watch 2), and the built-in accelerometer in the VR headset to provide a comparison to the gold-standard measure of indirect calorimetry.

The tertiary purpose of this study was to examine any changes to mood as a result of engagement in Flow and Boxing, as compared to the contemporary literature on VR exercise and mood [27,28].

## Methods

### Participants

Inclusion criteria for participants were (1) being 19-40 years of age; (2) self-reporting a minimum of 150 minutes of MPVA per week; (3) self-identifying as not being at increased risk for contracting COVID-19 or being a part of an immunocompromised population; and (4) being considered to have a minimal risk of an exercise-induced adverse outcome. To ensure participants with a broad range of VR experiences were recruited, recruitment documentation described the study as a “digital fitness experience” to avoid confounding any measurements collected. Participants became aware of the use of VR technology upon their receipt of the informed consent documentation before their first visit. Participants were recruited between September 2022 and December 2022 using a combination of social media postings, physical postings on the host university’s campus, and word of mouth.

### VR Headset and Game

Participants engaged with the VR fitness game Supernatural [29], which was played using the Oculus Quest 2 VR headset [30]. The Oculus Quest 2 uses a head-mounted display and 2 handheld controllers to provide an immersive VR experience for users. For this study, a 5-foot (1.5 m) by 5-foot space was marked onto the floor with tape to calibrate the in-headset play area boundaries (Multimedia Appendix 1). For each participant, the floor level was calibrated, and headset straps were adjusted to fit comfortably.

**Figure 1:**
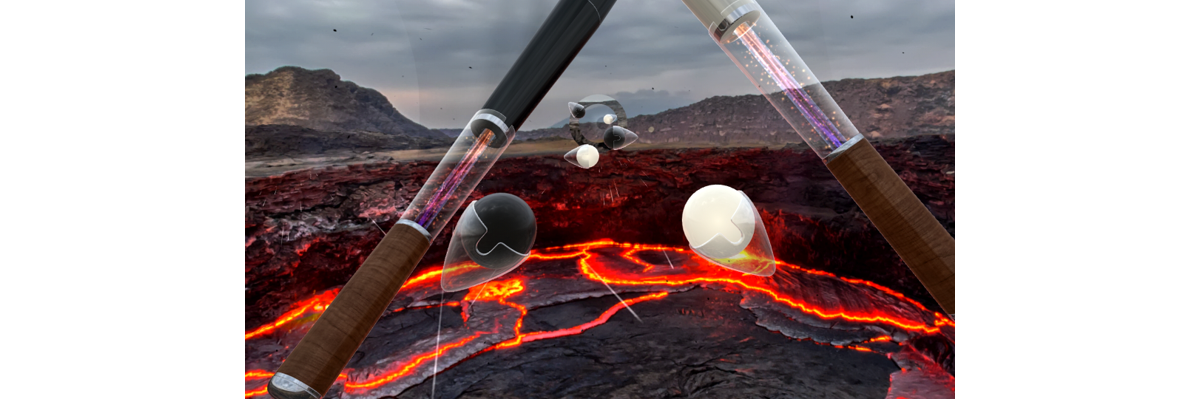
Supernatural Flow game mode.

**Figure 2:**
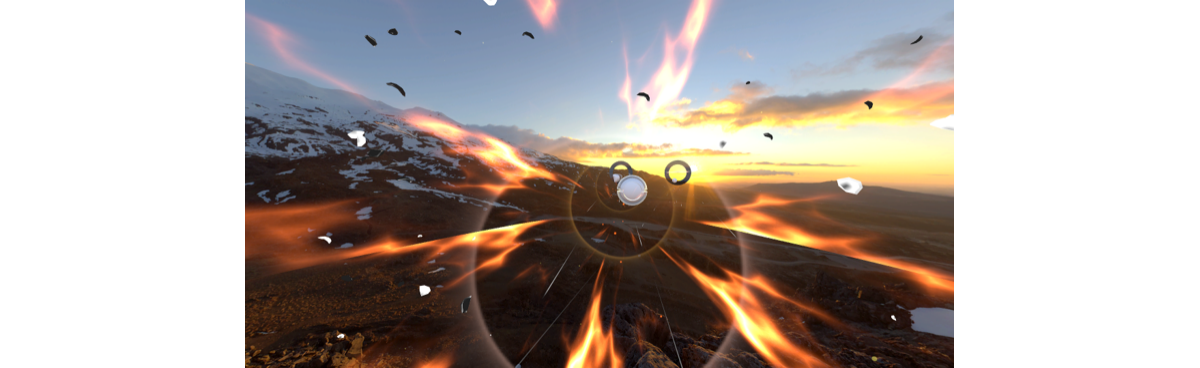
Supernatural Boxing game mode.

The Supernatural game has 2 exercise modes: Flow and Boxing. Both modalities cue participants to arm movements with color-coordinated orbs that have directional arrows to indicate movements to participants. There are also horizontal bars and triangles to encourage squatting, lunging, and the dodging of obstacles. During their first session, participants engaged in the workout mode Flow. Flow is an aerobic workout that involves both the upper and lower body and footwork in 360 degrees (Figure 1). Participants wield a virtual bat in each hand, striking targets in a variety of patterns and intensities. Lower-body movements are incorporated into each sequence, requiring participants to squat or lunge to hit some targets. During the second session, participants challenged a Boxing workout. Boxing requires participants to punch, uppercut, or swing through color-coordinated orbs and has horizontal and diagonal bars that participants have to maneuver their head and torso under and around (Figure 2). Note that still captures of Supernatural provide only an approximate impression of the actual, first-person in-app perspective of a Supernatural user on modern Meta Quest hardware.

### Experimental Overview

#### Overview

Interested participants contacted the researcher by email to arrange the initial session. Participants attended 2 sessions at the host institution which occurred a minimum of 24 hours apart. The first visit centered around the Supernatural Flow session, and the second visit was around the Supernatural Boxing session. This order was maintained for all participants. Participants were asked to abstain from engaging in MVPA for 12 hours before each session. During the first study visit, participants signed the informed consent document, completed a Get Active Questionnaire (GAQ [31]), had their anthropometric data collected, and completed a baseline survey assessing previous knowledge and exposure to VR. Before and after each session, participants’ mood states using the shortened version of the Profile of Mood States (POMS-SF [32]) were assessed. After initial surveys, participants were fitted with a heart rate (HR) monitor and the Oculus Quest 2 VR headset and then followed a standardized progression up to the measurement intensity workout until meeting the predetermined threshold for competency (see Zones of Competency section). Upon meeting the zone of competency, participants were fitted with the indirect calorimetry mask and headpiece, Apple Watch, and waist-worn accelerometer and completed the measurement session.

#### VR Game Stage Progression

Following initial surveys at each visit, participants watched a series of tutorial videos for the respective mode of the session (ie, Flow for session 1 and Boxing for session 2) which are available on Supernatural’s YouTube page. Participants then engaged in an in-game tutorial (approximately 5 minutes). Participants then proceeded to a low-intensity “Quick Hits” workout and a medium-intensity “Quick Hits” ramp-up workout, both approximately 5 minutes long, to further familiarize them with the mode. Participants who were able to meet the minimum zones of competency for each mode (as detailed in Zones of Competency section) proceeded to the measurement workout. Participants could repeat the “Quick Hits” workouts until the zones of competency were obtained. The measurement session was a medium-intensity workout between 14 and 17 minutes in length. All workouts were selected from a predetermined list of relatively similar-intensity workouts. In correspondence with Supernatural, the makeup of the different workouts within the same intensity is generally similar to one another in the range of motion, target origination, pace, and number of targets delivered. Participants chose the genre of music (eg, pop, rock, hip-hop, and electronic) they preferred, and a researcher would select a workout matching the description.

#### Zones of Competency

To advance to the measurement session, participants had to meet the predetermined zones of competency for each mode. Flow measures participant competency in accuracy (ie, percentage of targets hit) and power (ie, how fast targets were hit). Participants had to obtain a minimum of 92% accuracy to participate in the measurement session. Power was not used in the determination of participant readiness. Boxing measures participant competency in accuracy (ie, percentage of targets hit) and speed (ie, how fast targets were hit). Participants had to achieve a minimum of 94% accuracy in Boxing to progress to the measurement round. During preliminary testing, it was also determined that a minimum speed goal should be achieved to ensure participants were all working out at a similar intensity. Hence, a minimum score of 70% speed was determined to be sufficient, as this best reflected the lower end of the speed an average user would hit the targets at, according to correspondence with Supernatural.

### Instrumentation and Measurement

#### Primary Outcome: Energy Expenditure (Breath-by-Breath Oxygen Consumption)

A Parvo Medics TrueOne 2400 metabolic cart was used to assess oxygen consumption (VO_2_; ie, volume of oxygen) during both measurement sessions [33]. A 2-meter hose and a 3-meter hose were joined together using a polyvinyl chloride elbow joint and hose clamps to allow the hose to reach from the metabolic cart to loop suspended above the participant, then down to the participant to allow VO_2_ to be assessed with minimal interference during the testing (Multimedia Appendices 1 and 2). The metabolic cart was calibrated to room air and known gas concentrations before testing, followed by individual setup according to participants’ height, weight, age, and sex. Both objective VO_2_ max and relative VO_2_ max were collected. Relative VO_2_ max was also compared to age-predicted VO_2_ max. VO_2_ averages were recorded in 30-second intervals.

#### Secondary Outcomes

##### Energy Expenditure (Metabolic Equivalents and Caloric Expenditure)

Metabolic equivalents of task (METs) and caloric expenditure were assessed using the metabolic cart, an ActiGraph GTX3 triaxial waist-worn accelerometer, a Series 7 Apple Watch, and the built-in accelerometer on board the Oculus Quest 2 headset (as displayed through the Oculus Move in-headset app). Time spent in low-, moderate-, and high-intensity exercise was recorded by the waist-worn accelerometer. The waist-worn accelerometer was calibrated according to each participant’s age, height, weight, and sex. The Apple Watch and Oculus Move accounts were calibrated to a reference individual (female, height 181 cm, weight 78 kg) to avoid the need for an individual Apple and Oculus Move account for each participant. The Oculus Quest 2 boundaries were standardized to a 1.5 meter by 1.5 meter square.

##### Heart Rate

A Polar H10 HR monitor was used to measure average and maximum beats per minute (bpm) and the percentage of time spent in HR zones. Polar classifies HR into 5 zones by both levels of intensity and percentage of age-predicted maximum HR (HRmax). Specifically, these zones are categorized as zone 1: very light, 50%-60% HRmax; zone 2: light, 60%-70% HRmax; zone 3: moderate, 70%-80% HRmax; zone 4: hard, 80%-90% HRmax; and zone 5: maximum, 90%-100% HRmax [34]. The Polar H10 HR monitor was calibrated to a reference individual (female, height 181 cm, weight 78 kg) to avoid the need for an individual Polar account for each participant.

#### Tertiary Outcomes

##### Demographics

Participants’ age, sex, height, weight, waist circumference, and PA levels were recorded during the initial visit. Age, sex, and PA level were self-reported using a single item. Height, weight, and waist circumference were measured by a researcher.

##### Mood States

Participants completed surveys at the beginning and end of each session to assess potential changes in mood states, which were assessed with the POMS-SF [32]. Our specific subscales of interest were vigor (ie, lively, active, energetic, full of pep, and vigorous) and fatigue (ie, worn out, fatigued, exhausted, weary, and bushed), as these were thought to be the most receptive to participants’ exertion within our acute aerobic intervention and nonclinical sample [35,36]; however, the entire POMS-SF was completed by each participant.

### Data Handling and Analysis

#### Energy Expenditure Outcomes

Energy expenditure outcomes were analyzed descriptively (mean and SD) and assessed for normality. Winsorization [37] of any outliers was planned. An average of the objective VO_2_ max and relative VO_2_ max for Flow and Boxing sessions was calculated. The relative VO_2_ max was also calculated as a percentage of the age-predicted VO_2_ max. Accelerometer data were analyzed using the Freedson Adult MV3 cut points [38]. The accelerometer, Apple Watch, and Oculus Move data were compared to the metabolic cart data to determine the percentage difference between the measurement tools. Comparisons between modes (Flow and Boxing) and measurement modalities (ie, metabolic cart, waist-worn accelerometer, Apple Watch, and Oculus Move) were compared for the 21 complete data sets (n=11, 52% male individuals) using paired sample 2-tailed *t* tests using a Bonferroni correction (ie, α=.0125). Missing data were excluded case-wise.

#### Psychological Outcomes

Changes in mood state (ie, vigor and fatigue subscales) from pre- to postsession for both Flow and Boxing modalities were assessed using paired 2-tailed *t* tests.

#### Sample Size Determination

To be sufficiently powered to do a sex-specific subanalysis, we aimed to collect at least 20 full data sets (ie, 10 male and 10 female individuals). This number was determined based on an investigation of previous energy expenditure and VR fitness publication recruitment numbers and was deemed appropriate to capture sex-specific differences in energy expenditure outcomes [39-41]. Sex-specific subanalyses were only performed for the energy expenditure outcomes, as the sample was deemed to be too small to capture small-to-medium–sized effects on psychological outcomes.

### Ethical Considerations

This prospective, single-group experimental study was approved by the host University of Victoria's research ethics board (22-0213), and all participants provided written, informed consent before their involvement in the study and for inclusion in the publication of any research findings, as indicated by the Declaration of Helsinki.

## Results

A CONSORT (Consolidated Standards of Reporting Trials)-eHealth checklist for this study can be found in Multimedia Appendix 3.

### Participant Characteristics

A total of 12 male and 12 female individuals who met inclusion criteria and provided informed consent were recruited for this study. One male participant dropped out before beginning the Flow data collection due to issues with the VR environment. Overall, 2 female participants completed the Flow session but not the Boxing session due to factors unrelated to the study. A total of 23 (n=11, 48% male) participants completed the Flow session, and a total of 21 participants (n=11, 52% male) completed the Boxing session.

Participant demographics are presented in Table 1. The average age of participants was 25.42 (SD 3.25) years. Participants reported participating in MVPA 4.71 (SD 1.62) days a week for an average of 72.38 (SD 39.04) minutes per session, classifying our participants as active [3]. Our participant pool was very naive to VR and VR fitness, however, with 96% (n=23) reporting no previous familiarity with VR fitness products before study participation. There were no outliers in the data set.

**Table 1:** Participant demographics.

Characteristic	Male participants (n=12), mean (SD)	Female participants (n=12), mean (SD)
Age (years)	25.5 (3.1)	25.2 (3.0)
Height (cm)	178.1 (9.9)	166.6 (7.9)
Weight (kg)	76.7 (14.1)	64.36 (7.9)
Waist circumference (cm)	85.5 (6.2)	76.0 (5.1)

### Workout Characteristics

The average Flow workout was 15.48 (SD 1.31) minutes in duration and was completed with 94.57% (SD 2.35%) accuracy and 85.52% (SD 5.27%) power. The average Boxing workout was slightly longer than Flow at 16.91 (SD 1.51) minutes in length, with 96.67% (SD 1.77%) accuracy and 78.86% (SD 7.89%) speed.

### Energy Expenditure

#### Oxygen Consumption

Objective VO_2_ max, as measured by the metabolic cart, was 1.98 (SD 0.44) L/min for Flow, 1.88 (SD 0.45) L/min for Boxing, and 1.93 (SD 0.44) L/min overall. Relative VO_2_ max was 28.52 (SD 5.39) mL/kg/min for Flow, 26.70 (SD 5.79) mL/kg/min for Boxing, and 27.61 (SD 5.60) mL/kg/min overall. There was a significant difference between Flow and Boxing for both objective (mean difference [M_diff_]=0.14, 95% CI 0.05-0.24; *P*=.006) and relative VO_2_ max (M_diff_=2.05, 95% CI 0.53-3.56; *P*=.01). The percentage of age-predicted VO_2_ max was 59.39% (SD 11.75%) for Flow, 55.42% (SD 12.45%) for Boxing, and 57.41% (SD 12.12%) overall. There was no difference between Flow and Boxing with respect to the percentage of age-predicted VO_2_ max (M_diff_=4.20%, 95% CI 1.34%-7.05%; *P*=.006).

With respect to sex-specific differences, a significant difference was observed for objective VO_2_, with male individuals demonstrating a significantly higher objective VO_2_ for Flow than female individuals (M_diff_=0.46, 95% CI 0.13-0.78; *P*=.009). Sex differences for objective VO_2_ for Boxing were not statistically significant (M_diff_=0.36, 95% CI –0.02 to 0.74; *P*=.06). No significant differences between sexes were revealed for any other VO_2_ outcome (*P*>.05).

#### Outcomes for METs

METs were collected by both the metabolic cart and the waist-worn accelerometer. The metabolic cart recorded average METs to be 8.15 (SD 1.54) for Flow, 7.63 (SD 1.66) for Boxing, and 7.89 (SD 1.60) overall, while the waist-worn accelerometer recorded averages as 4.31 (SD 0.56) for Flow, 4.78 (SD 0.57) for Boxing, and 4.55 (SD 0.65) overall. For data recorded by the metabolic cart, there was a significant difference between Flow and Boxing modes (M_diff_=0.58, 95% CI 0.15-1.02; *P*=.01). There was a significant difference between the values recorded by the metabolic cart and the waist-worn accelerometer for both Flow (M_diff_=3.69, 95% CI 3.12-4.25; *P*<.001) and Boxing (M_diff_=2.84, 95% CI 2.21-3.47; *P*<.001), with the accelerometer reporting a percent of metabolic cart reading of 45.26% (SD 14.55%) for Flow, 56.03% (SD 13.80%) for Boxing, and 50.64% (SD 15.05%) overall.

With respect to sex-specific differences, no significant differences were revealed for METs as assessed by either the metabolic cart or waist-worn accelerometer (*P*>.05). Oxygen consumption and metabolic equivalent data are given in Table 2.

**Table 2:** Oxygen consumption (VO2) and metabolic equivalent of task (MET) data.

Energy expenditure outcome	Flow, mean (SD)	Boxing, mean (SD)	Overall, mean (SD)
Objective VO_2_ (L/min)	1.98 (0.44)^a^	1.88 (0.45)^a^	1.93 (0.44)
Relative VO_2_ (mL/kg/min)	28.52 (5.39)	26.70 (5.79)	27.61 (5.60)
Age-predicted VO_2_ max^b^ (%)	59.39 (11.75)	55.42 (12.45)	57.41 (12.12)
METs (metabolic cart)	8.15 (1.54)^a^	7.63 (1.66)^a^	7.89 (1.60)
METs (accelerometer)	4.31 (0.56)	4.78 (0.57)	4.55 (0.65)

^a^Values represent a significant difference between modes (ie, Flow and Boxing).

^b^VO_2_ max: maximal oxygen consumption.

***Calories***

The estimated caloric expenditure of the metabolic cart, waist-worn accelerometer, Apple Watch, and Oculus are presented in Table 3. For Flow, both the accelerometer (M_diff_=79.25, 95% CI 68.30-90.20; *P*<.001) and Oculus Move (M_diff_=30.79, 95% CI 12.84-48.74; *P*=.002) were found to be significantly different from the metabolic cart, while the Apple Watch (M_diff_=–4.83, 95% CI –18.30 to 8.66; *P*=.47) was not. Similarly, for Boxing, both the accelerometer (M_diff_=70.56, 95% CI 56.59-84.53; *P*<.001) and Oculus Move (M_diff_=62.89, 95% CI 45.52-80.26; *P<*.001) were found to be significantly different from the metabolic cart, while the Apple Watch (M_diff_=–14.24, 95% CI –32.22 to 3.73; *P*=.11) was not.

**Table 3:** Caloric expenditure data.

Device	Flow (kcal)		Boxing (kcal)		Overall (kcal)	
	Mean (SD)	% Metabolic cart	Mean (SD)	% Metabolic cart	Mean (SD)	% Metabolic cart
Metabolic cart	151.22 (35.52)	N/A^a^	159.76 (38.829)	N/A	155.49 (36.95)	N/A
Accelerometer	70.60 (22.34)^b^	45.26^b^	89.20 (28.892)^b^	56.03^b^	79.90 (27.12)^b^	50.46^b^
Apple Watch	155.41 (24.87)	107.56	174.00 (39.607)	112.34	164.71 (33.59)	109.95
Oculus Move	121.27 (21.98)^b^	82.88^b^	102.00 (17.914)^b^	63.75^b^	111.63 (22.06)^b^	73.32^b^

^a^N/A: not applicable.

^b^Values represent a significant difference from the metabolic cart (*P*≤.0125).

#### Heart Rate

**Figure 3:**
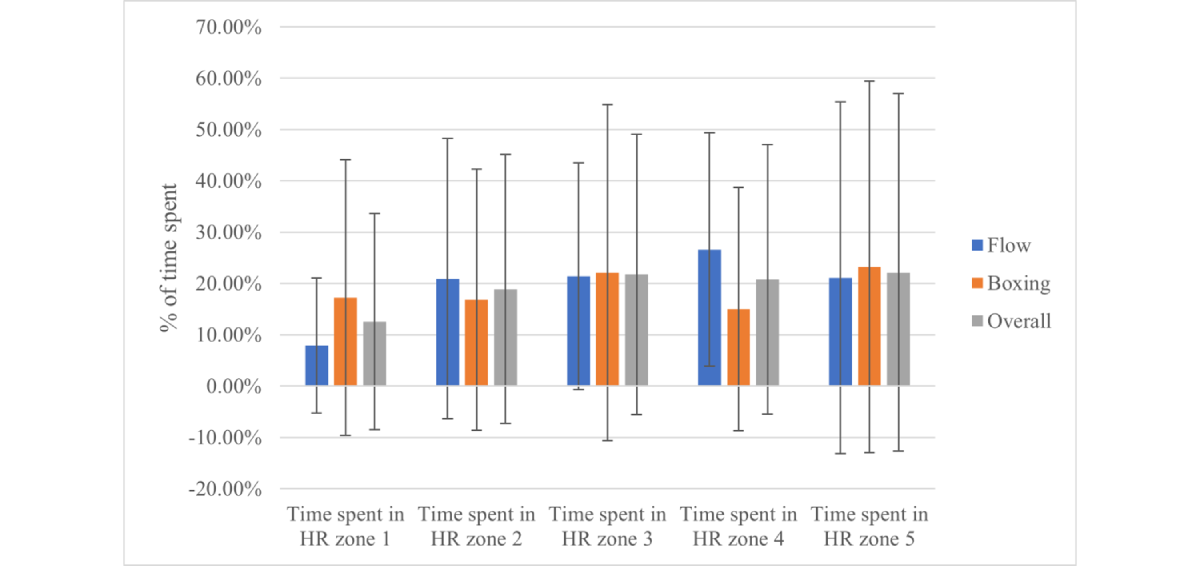
Time spent in heart rate zones. Error bars represent the SD. HR: heart rate.

The average HR for Flow was 151.13 (SD 23.12) bpm, and HRmax was 169.39 (SD 20.04) bpm. The average HR for Boxing was 143.29 (SD 26.36) bpm, with an HRmax of 161.43 (SD 26.36) bpm. Flow demonstrated a significantly higher average HR (M_diff_=8.43, 95% CI 3.45-13.40; *P*=.002) and HRmax (M_diff_=8.38, 95% CI 3.12-13.64; *P*=.003) when compared to Boxing. The overall average HR across both modalities was 147.21 (SD 23.12) bpm, and the average overall HRmax was 165.41 (SD 20.04). Sex-specific differences in HR were observed for Boxing average HR (M_diff_=25.61, 95% CI 1.99-49.23; *P*=.04) and HRmax (M_diff_=25.53, 95% CI 4.04-47.01; *P*=.02), with male individuals demonstrating a higher HR during Boxing. Time spent in HR zones is presented in Figure 3.

#### Changes in Mood States

Between baseline and postsession 1, on average, participants reported that they felt more “lively” (M_diff_=0.46, 95% CI 0.50-0.86; *P*=.03), more “active” (M_diff_=0.67, 95% CI 0.32-1.07; *P*<.001), more “full of pep” (M_diff_=0.57, 95% CI 0.14-0.99; *P*=.01), and more “vigorous” (M_diff_=0.48, 95% CI 0.90-0.87; *P*=.02). Between presession 2 and postsession 2, on average, participants reported feeling more “active” (M_diff_=0.76, 95% CI 0.36-1.17; *P*<.001), more “full of pep” (M_diff_=0.38, 95% CI 0.02-0.75; *P*=.04), and more “vigorous” (M_diff_=0.52, 95% CI 0.18-0.87; *P*=.004). No item on the fatigue subscale of the POMS-SF changed significantly for either session 1 or 2.

## Discussion

VR fitness games have seen an increase in popularity as a mode of PA in recent years [7]. However, variability among game demands and objectives, along with difficulties in measuring energy expenditure with gold standard assessments (ie, indirect calorimetry), have limited our understanding of the intensity of these games [19], in turn limiting recommendations involving this mode of PA with respect to MVPA guidelines. Hence, the primary aim of this study was to examine the energy expenditure of one of the most popular VR fitness games, Supernatural. Specifically, we assessed the energy expenditure of a session of medium intensity for both the Flow and Boxing modes of Supernatural using indirect calorimetry. We also examined how other popular measures of energy expenditure compared relative to indirect calorimetry.

For Flow, average relative VO_2_ max was 28.52 (SD 5.39) mL/kg/min, which translated to approximately 8.2 (SD 1.54) METs, classifying it as vigorous intensity (ie, >6 METs) [42]. Compared to other forms of PA, Flow was akin to climbing stairs (8.0 METs) or general circuit training (8.0 METs) [43]. Moreover, the percent of age-predicted VO_2_ max (mean 59.39%, SD 11.75%) points to the potential use of this mode of Supernatural within a VO_2_ training program [44]. Notably, only objective VO_2_ was found to be significantly different between male and female participants, which suggests that this mode of PA demonstrates a significant sex difference in objective energy expenditure. This finding is likely due to the body weight nature of the game (ie, movements were relative to participants’ own body weight), as this significant difference was not evident when examining the relative VO_2_ values or METs (ie, accounting for participants’ weight). This is encouraging, suggesting that young, active individuals engaging in a bout of Supernatural Flow should receive a relatively similar aerobic workout, independent of sex. This indicates that during a flow session, participants were averaging a high enough percentage of VO_2_ to potentially improve maximum oxygen uptake.

For Boxing, average relative VO_2_ max was 26.70 (SD 5.79) mL/kg/min, which translated to approximately 7.6 (SD 1.66) METs, also classifying it as vigorous intensity (ie, >6 METs) [42]. Compared to other modes of PA, Boxing was higher than high-impact aerobics (7.3 METs) and close to sparring while boxing (7.8 METs) [43]. Interestingly, both the Flow and Boxing modes demonstrated a higher energy expenditure than “activity-promoting video or arcade game (eg, Exergaming and Dance Dance Revolution), vigorous effort” (7.2 METs), which further speaks to the heterogeneity in demands among available VR fitness games [45]. For example, the lower average energy expenditure of the Boxing mode may be due to the difference in physical demands between modes, with Flow incorporating more frequent multimuscle group movements (eg, squats and arm swings) than Boxing, which consists primarily of slips and punches. Similar to Flow, there was a trend in objective VO_2_ between sexes (*P*=.06) favoring male individuals but no significant differences in any other VO_2_ outcome or METs, for the Boxing session. Furthermore, while the objective VO_2_ max and METs were significantly lower for Boxing than Flow, the data suggests that individuals were still exercising at a high enough percentage of VO_2_ to improve maximal oxygen uptake [46]. Hence, Boxing also has implications as a candidate for use in aerobic training programs, independent of sex.

Given the lack of research comparing indirect calorimetry to device-based measures when examining VR fitness, we also aimed to compare the findings from the metabolic cart to that of a triaxial waist-worn accelerometer, an Apple Watch, and the Oculus Move. On average, the accelerometers underestimated the energy expenditure of the metabolic cart by approximately 65% (SD 14.55%) for Flow and approximately 45% (SD 13.8%) for Boxing. This discrepancy and variability were somewhat surprising, given the relative reliability and validity of this particular accelerometer in assessing aerobic PA (eg, running and walking) [47]. However, this underestimation is likely due to the placement of the accelerometer (ie, waist-worn), which is unlikely to capture the full range of dynamic upper and lower body movements characteristic of a VR fitness game. Notably, several other studies have used waist-worn accelerometers within VR research [25,26], though these studies have examined time spent in MVPA rather than an energy expenditure outcome (eg, METs). Despite this, an underrepresentation of MVPA as captured by accelerometry still appears to be evident; Sousa et al [26] reported an average of 4.10 (SD 4.93) minutes of MVPA for a 20-minute VR fitness session (approximately 21%), while Giakoni-Ramirez and colleagues [25] reported an average of 3.57 minutes of MVPA for a 9-minute “intermediate” VR fitness session (approximately 40%). Hence, our findings suggest that the use of accelerometers during this form of PA be used cautiously.

Compared to the accelerometer, the Apple Watch was relatively more accurate, estimating approximately 108% (SD 24.88%) and approximately 112% (SD 25.41%) of the metabolic cart for Flow and Boxing, respectively. This may be due to the wrist-worn placement of the Apple Watch, which makes it more sensitive to the movements of the VR fitness games. Notably, there was a considerable degree of variability within the Apple Watch measures, ranging from a 28% underestimation to a 56% overestimation of caloric expenditure. Our findings are consistent with previous validation work using the Apple Watch to measure MVPA and energy expenditure [48]. Work by Bai and colleagues [49] does support the validity of the Apple Watch as a measure of MVPA; importantly, however, their work uses a waist-worn accelerometer as the criterion measure, which limits the interpretability of their findings in a VR fitness context. Hence, while our results support the relative accuracy of the Apple Watch, its usefulness as a precise measure of energy expenditure during VR fitness is limited.

On average, the Oculus Move accelerometer underestimated the energy expenditure of the VR fitness games measured by the metabolic cart by 27% (SD 23.1%) and 44% (SD 12.48%) for Flow and Boxing, respectively. Like the waist-worn accelerometer, the placement of the accelerometer within the Oculus headset may have impacted the accuracy of its measurements. Similar to the other measures of energy expenditure, the use of this outcome as a measure of energy expenditure is limited.

Lastly, preliminary comparisons of pre-post VR fitness measurement session mood states revealed reported changes in mood that are consistent with the beneficial changes we would expect from a bout of moderate-intensity PA [36] and with previous VR exergaming research [28,35]. These changes provide preliminary evidence for the positive mental health benefits of even single sessions of VR fitness, which is encouraging given the relationship between positive affect and adherence to PA [50].

Though our study contained many strengths, such as a gold-standard measure of energy expenditure (ie, indirect calorimetry) and a relatively homogeneous sample, there are limitations to our work. One limitation of this study is that all participants were naive to VR fitness. This may have resulted in a variable amount of energy expended compared to someone who regularly engages in VR fitness. Although we implemented a strict threshold for inclusion in the measurement session, discrepancies in the final accuracy and power scores, along with direct observation of participants, suggest that some participants spent more or less energy adjusting to the difficulty of the measurement session workout. In other words, the final score across participants suggests that some struggled more than others in acclimating to the difficulty of the measurement session. Further, all participants were active individuals (ie, meeting the weekly PA guidelines). As a result, the measured metabolic and cardiorespiratory responses to the measurement session may not be reflective of the average new user.

Both the Flow and Boxing medium-intensity modes of Supernatural demonstrated relatively high energy expenditures (8.2, SD 1.54 METs, and 7.6, SD 1.66 METs, respectively), classifying as vigorous-intensity PA (ie, >6.0 METs) [42]. Device-based measures of energy expenditure varied considerably both between participants and when compared to the metabolic cart results. Hence, caution should be used when using and interpreting device-based measures of energy expenditure within VR fitness games, including Supernatural. This study also provides preliminary evidence to support the physical and mental health benefits of engaging in VR fitness games like Supernatural. These findings are encouraging, given the increasing popularity and accessibility of VR fitness games as a means of achieving MVPA. For individuals who are interested in being physically active at home or are unable to access traditional forms of exercise, VR fitness presents a potential supplement or alternative to achieving the recommended levels of MVPA.

## Appendix

Multimedia Appendix 1 Experimental setup in action detailing the Oculus Quest 2 VR headset, metabolic cart apparatus, Apple Watch 2, and ActiGraph waist-worn accelerometer (located beneath model’s shirt).

Multimedia Appendix 2 Experimental setup including Oculus Quest 2 VR headset and metabolic cart headgear and mouthpiece.

Multimedia Appendix 3 CONSORT (Consolidated Standards of Reporting Trials)-eHealth Checklist.

## Abbreviations

CONSORT: Consolidated Standards of Reporting Trials
GAQ: Get Active Questionnaire
HR: heart rate
HRmax: maximum heart rate
Mdiff: mean difference
MET: metabolic equivalent of task
MVPA: moderate to vigorous intensity physical activity
PA: physical activity
POMS-SF: shortened version of Profile of Mood States
VO2 max: maximal oxygen consumption.
VO2: oxygen consumption.
VR: virtual reality

## Data Availability

The data sets generated and analyzed during this study are not publicly available due to privacy restrictions in accordance with the study sponsor but are available from the corresponding author on reasonable request.
