# Supplementary figures and images for: Examining and Comparing the Energy Expenditure of Two Modes of a Virtual Reality Fitness Game (Supernatural): Indirect Calorimetry Study
Source: JMIR Serious Games. 2024 Jun 4;12:e53999. doi: 10.2196/53999 (PMC11185914; doi:10.2196/53999)

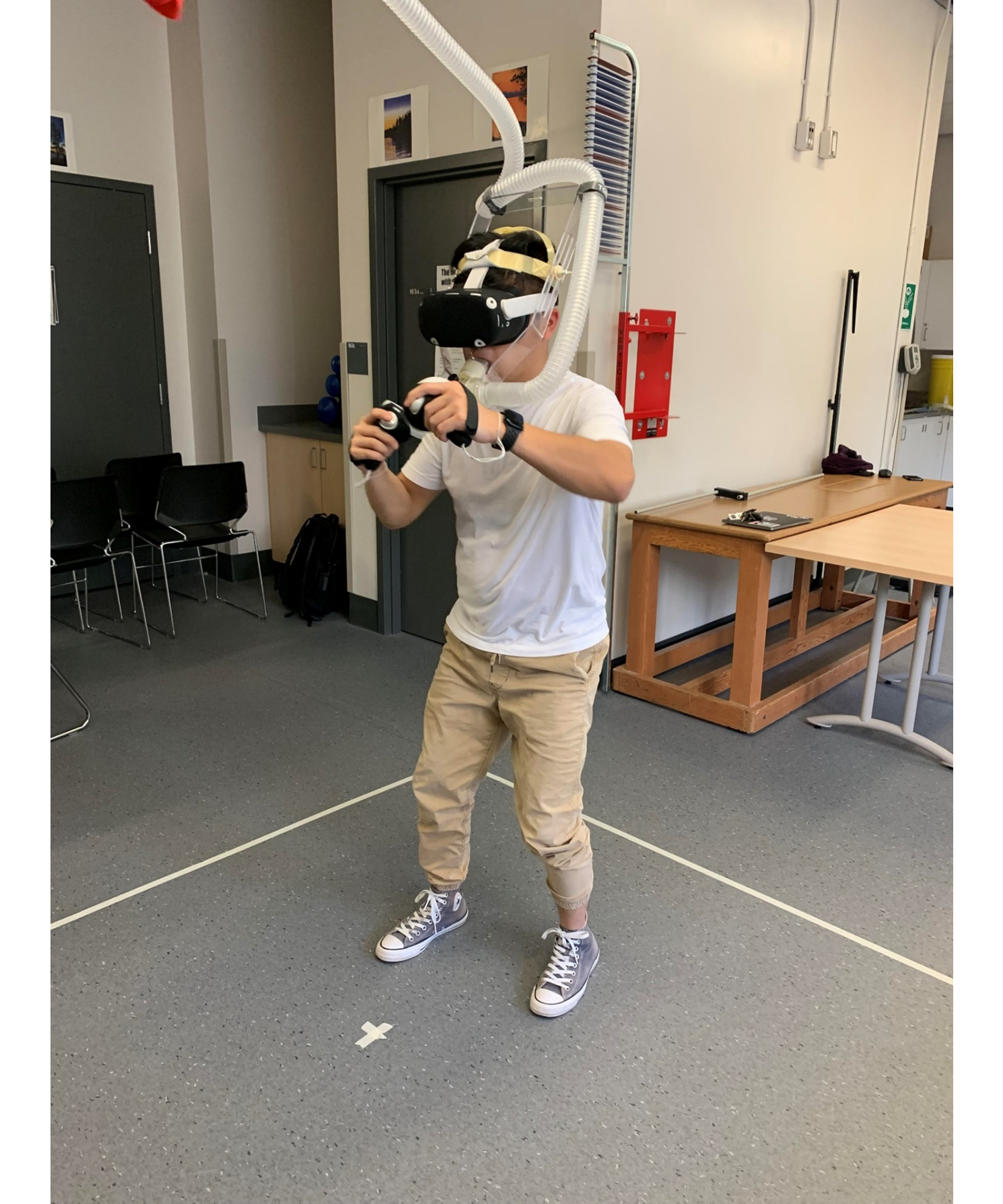

Supplement: Multimedia Appendix 1 [file games_v12i1e53999_app1.png]

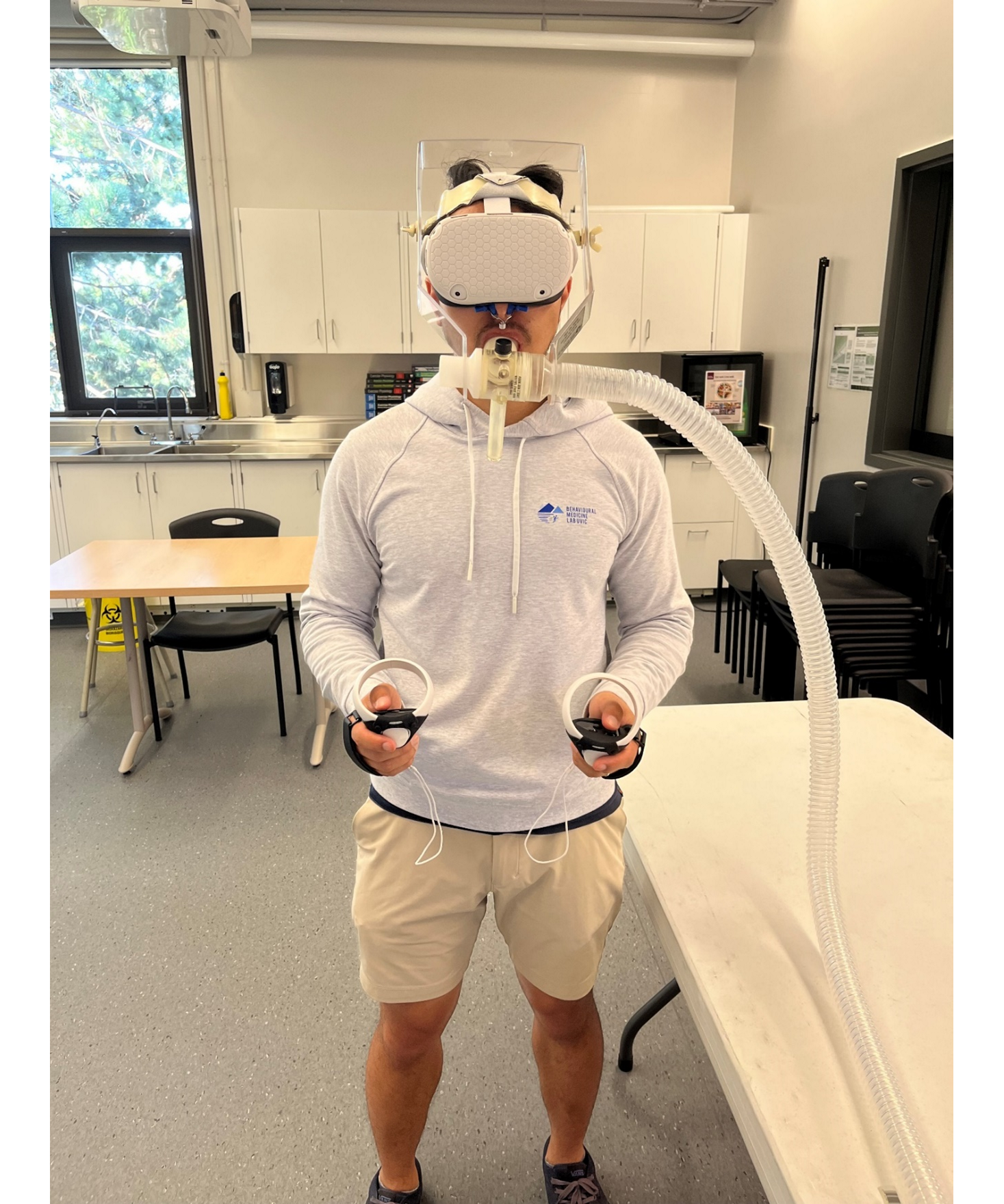

Supplement: Multimedia Appendix 2 [file games_v12i1e53999_app2.png]
